# Unilateral Brachial Plexus Lesion Impairs Bilateral Touch Threshold

**DOI:** 10.3389/fneur.2019.00872

**Published:** 2019-08-13

**Authors:** Bia Lima Ramalho, Maria Luíza Rangel, Ana Carolina Schmaedeke, Fátima Smith Erthal, Claudia D. Vargas

**Affiliations:** ^1^Laboratory of Neurobiology of Movement, Institute of Biophysics Carlos Chagas Filho, Federal University of Rio de Janeiro, Rio de Janeiro, Brazil; ^2^Laboratory of Neuroscience and Rehabilitation, Institute of Neurology Deolindo Couto, Federal University of Rio de Janeiro, Rio de Janeiro, Brazil; ^3^Laboratory of Neurobiology II, Institute of Biophysics Carlos Chagas Filho, Federal University of Rio de Janeiro, Rio de Janeiro, Brazil

**Keywords:** Semmens-Weinstein monofilaments, sensory threshold, brachial plexus neuropathy, impairment, light touch sensation, deafferentation, uninjured

## Abstract

Unilateral brachial plexus injury (BPI) impairs sensory and motor functions of the upper limb. This study aimed to map in detail brachial plexus sensory impairment both in the injured and the uninjured upper limb. Touch sensation was measured through Semmes-Weinstein monofilaments at the autonomous regions of the brachial plexus nerves, hereafter called points of exclusive innervation (PEIs). Seventeen BPI patients (31.35 years±6.9 SD) and 14 age-matched healthy controls (27.57 years±5.8 SD) were tested bilaterally at six selected PEIs (axillary, musculocutaneous, median, radial, ulnar, and medial antebrachial cutaneous [MABC]). As expected, the comparison between the control group and the brachial plexus patients' injured limb showed a robust difference for all PEIs (*p* ≤ 0.001). Moreover, the comparison between the control group and the brachial plexus uninjured limb revealed a difference for the median (*p* = 0.0074), radial (*p* = 0.0185), ulnar (*p* = 0.0404), and MABC (*p* = 0.0328) PEIs. After splitting the sample into two groups with respect to the dominance of the injured limb, higher threshold values were found for the uninjured side when it occurred in the right dominant limb compared to the control group at the median (*p* = 0.0456), radial (*p* = 0.0096), and MABC (*p* = 0.0078) PEIs. This effect was absent for the left, non-dominant arm. To assess the effect of the severity of sensory deficits observed in the injured limb upon the alterations of the uninjured limb, a K-means clustering algorithm (k = 2) was applied resulting in two groups with less or more severe sensory impairment. The less severely affected patients presented higher thresholds at the median (*p* = 0.0189), radial (*p* = 0.0081), ulnar (*p* = 0.0253), and MABC (*p* = 0.0187) PEIs in the uninjured limb in comparison with the control group, whereas higher thresholds at the uninjured limb were found only for the median PEI (*p* = 0.0457) in the more severely affected group. In conclusion, an expressive reduction in touch threshold was found for the injured limb allowing a precise mapping of the impairment caused by the BPI. Crucially, BPI also led to reduced tactile threshold in specific PEIs in the uninjured upper limb. These new findings suggest a superordinate model of representational plasticity occurring bilaterally in the brain after a unilateral peripheral injury.

## Introduction

Brachial plexus injury (BPI) affects the sensory and motor functions of the upper limb to varying degrees resulting in complex patterns of sensorimotor dysfunction, often with very poor prognosis (1, 2). Affecting predominantly young male subjects [20–29 years, 89% male—(1)], BPI has major impacts on psychosocial well-being and quality of life (3, 4). A key aspect is that BPI often leads to loss of at least part of the upper limb movements (5) thus putatively compromising movements such as directional reaching, grasping and skilled manipulative movements with the upper limb. This is due not only to the loss of motor impairment, but also to the loss of sensation, known to be of particular importance in manipulative movements (6) [review in (7)]. Although most research in BPI has focused on motor impairment, relatively little has been done to further our understanding of its associated sensory dysfunction, despite its potential to inform both to clinicians and researchers about its severity and degree of impairment as well as to help improve strategies to increase the functionality of the upper limb.

Furthermore, current investigations of sensory dysfunction after a peripheral deafferentation consider the sensory deficit evaluation only in the injured limb and are often directed to a limited portion of the affected body surface (8–14). Given the heterogeneous nature of BPIs, it is important to make a complete investigation of all nerve territories of the BP, especially in its autonomous zones (2), corresponding to areas of skin in which each single nerve can be better assessed (15–17).

Although usually not evaluated after a peripheral nerve injury, changes in sensory function affecting the uninjured limb have been found in a variety of deafferentation models such as nerve block (18), nerve injury (19), amputation (20, 21), and in burned patients (22, 23). These changes have generally been interpreted as being the result of central nervous system adaptations occurring after the deafferentation.

Considering the lack of studies investigating the uninjured limb and possible sensory changes resulting from a BPI lesion, this study aimed to evaluate the sensory thresholds in BPI patients using Semmes-Weinstein monofilaments (SWM). We expected that a complete investigation of sensory thresholds in both upper limbs would allow not only the identification of the pattern of lesion-induced loss of sensation in the most affected limb but also reveal possible sensory deficits in the uninjured upper limb. This might expand the current knowledge on sensory changes after peripheral nerve lesions, providing novel approaches to clinical evaluation and also opening up new possibilities for the study of central reorganization after peripheral injury.

## Methods

### Participants

Seventeen BPI patients (2 females) with a mean age of 31.35 years ± 6.9 SD (19–40), were recruited at the Institute of Neurology Deolindo Couto (INDC-UFRJ). Fifteen patients were right handed (1 left handed and 1 ambidextrous) (24). Inclusion criteria were age equal to or above 18 years, preserved ability to communicate and unilateral traumatic BPI (any level and severity, pre- or post- nerve surgery, see Table 1), diagnosed through clinical and complementary exams such as electromyography and magnetic resonance imaging. Exclusion criteria were a previous history of primary or secondary central and peripheral nervous system disease. Participants were consecutively recruited between the years 2014 and 2015.

**Table 1:** BPI patient characteristics and time between injury and surgery and time between injury and the assessment.

**Id**	**Hand**.	**Lesion**	**Side**	**T1**	**Surgical procedures**	**T2**
BPI01	R	S, M, I	R	x	x	5.3
BPI02	R	S, M, I	R	6.1	C5 graft and unsp. NT	15.2
BPI03	A	S, M, I	L	3.3/4.2	Int. to Musc. NT + Sup. NN	28.2
BPI04	L	S, M, I	L	3	Int. to Musc. NT + Acc. to Sup. NT	24
BPI05	R	S, M, I	L	12.2	C5, C6, and C7 NN	13.2
BPI06	R	S, M, I	L	8.4	Acc. to Sup. NT	36.1
BPI07	R	S	R	6.1	Ul. to Musc. NT + Acc. to Sup. NT + Rad. to Axi. NT	45.7
BPI08	R	S, M	R	4.9	Ul. to Musc. NT + Acc. to Sup. NT	7.7
BPI09	R	S, M	L	11.7	Ul. to Musc. NT	12.9
BPI10	R	I	R	x	x	3
BPI11	R	S, M	L	11.3	Ul. to Musc. NT	17.6
BPI12	R	S, M, I	L	5.6	Int. to Musc. NT + unsp. NN and neuroma dissection	7.5
BPI13	R	S, M	R	6/10.4	Ul. to Musc. NT + Acc. to Sup. NT	12.4
BPI14	R	S, M	L	4.6/10.7	Ul. to Musc. NT + unsp. NN	15
BPI15	R	S, M, I	L	x	x	14.1
BPI16	R	S, M, I	L	3	Int. to Musc. NT + Acc. to Sup. NT	5.5
BPI17	R	S, M, I	R	11.8/13.8	Int. to Musc. NT + Acc. to Sup. NT	18.4

*Hand., handedness; R, right; L, left; A, ambidextrous; S, superior; M, middle; I, inferior; NT, nerve transfer; Int. to Musc, intercostal to musculocutaneous; Acc. to Sup., accessory to suprascapular; Ul. to Musc., ulnar to musculocutaneous; Rad. to Axil., radial to axillary; NN, nerve neurolysis; Sup., suprascapular nerve; unsp., unspecified procedures; T1, time between the injury and the surgery in months; T2, time between the injury and the sensory assessment in months*.

Eighteen healthy right-handed participants were also evaluated, and from this sample fourteen age-matched healthy participants were included in the analysis (27.57 years ± 5.8 SD). The remaining four participants were excluded based on k-means clustering applied to homogenize the sample (see Statistics).

Before the evaluation, all participants were asked whether they felt comfortable enough to be evaluated. They were then informed about the experimental procedures and provided written informed consent to participate in the study, which was approved by the local ethics committee (Institute of Neurology Deolindo Couto—UFRJ, Brazil) and was in accordance with the declaration of Helsinki.

### Semmes-Weinstein Monofilaments Assessment

In order to assess touch thresholds of the upper limb, a set of 20 Semmes-Weinstein Monofilaments (SWM, Bioseb, Vitrolles, France) were used. SWM are classified by the necessary force in grams to bend them against the skin, ranging from 0.007 to 160 g or, as expressed in log (10 × F; with F = force in milligrams), 1.84 to 6.20. The force values for each monofilament after the assessment using an analytical balance (Shimadzu Corp., Kyoto, Japan) were slightly different from the values specified by the manufacturer (see Supplementary Material). We decided to use the values we found rather than those of the manufacturer. Values for each monofilament are displayed in Supplementary Table 1.

**Figure 1:**
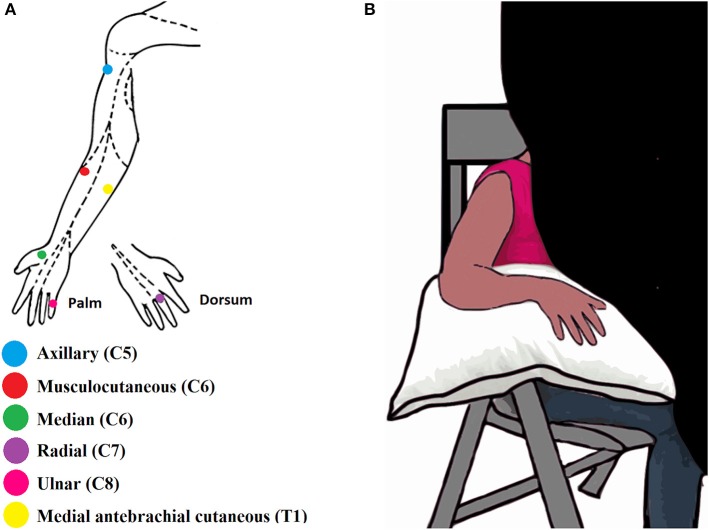
**(A)** Illustration of the six Points of Exclusive Innervation (PEIs) in the upper limb. **(B)** Illustration of the experimental setup.

Six stimulation points, called hereafter points of exclusive innervation (PEIs), were identified to perform the sensory test. The PEIs were within the five dermatomes (C5-T1) (25) of the BP and corresponded to the autonomous zones of six BP nerves: axillary, musculocutaneous, median, radial, ulnar and medial antebrachial cutaneous (MABC) (15) (Figure 1A).

During the assessment, participants sat in a chair in a quiet room together with the experimenters BR and AS. The upper limb to be tested rested on a pillow. Each limb was assessed separately. A black curtain blocked the volunteer's view of the assessed limb (Figure 1B). Both experimenters were trained to conduct the assessment the same way and while one experimenter was applying the filaments the other one was taking notes about the evoked sensations and the threshold values. The experimenters were not blind regarding the subject's condition since all the patients had the injured arm with some degree of paralysis and hypotrophy. The uninjured upper limb of the BPI patients was assessed first, or the right upper limb, in the case of healthy volunteers. The order of PEI stimulation was pseudo-randomized for each upper limb. For each PEI, monofilaments were applied in ascending order—from the thinnest to the thickest. The thinnest filament detected by the participant in 3 out of 5 applications was considered as the tactile threshold for that PEI (26). All the procedures were explained and demonstrated to the participants in advance. The intervals between each stimulation were arbitrary to avoid the learning of the stimulation sequence and, consequently, false positives. When the participant was not able to feel even the thickest filament (6.20, see Supplementary Table 1) his touch threshold was established at this filament (6.20).

### Statistical Analysis

Threshold values for each participant were log transformed (10 × force in mg—see Supplementary Table 1). For each PEI, the threshold values were described as medians.

#### K-means Clustering

Both for the control and the patients' group we used k-means cluster analysis (27) to assign individuals to clusters based on their threshold values for the six PEIs. The k-means algorithm partitions the sample into k clusters based on variables of interest. The algorithm uses a heuristic to find centroid seeds for k-means clustering and then computes the squared Euclidean distance from each observation to each centroid, assigning each observation to its closest centroid. The goal of this procedure is to minimize the within-cluster variance and maximize the between-cluster variance.

The D'agostino-Pearson test was then used to assess normality of the groups. Since data from some PEIs did not respect Gaussian distribution, non-parametric tests were applied.

#### Control Group

For setting the control group, the K-means clustering algorithm was applied and resulted in a cluster comprising 14 participants (four participants were excluded from further analyses). The comparison between each PEI with its counterpart in the same control participant (right arm vs. left arm) was performed by means of the Wilcoxon signed-rank test. Since the comparison between right and left upper PEIs revealed no difference (axillary, *p* = 0.5000; musculocutaneous, *p* = 0.7127; median, *p* = 0.3523; radial, *p* = 0.3493; ulnar, *p* = 1; MABC, *p* = 0.2123), the mean between the corresponding right and left PEIs from both upper limbs was calculated. This procedure was performed to establish a single threshold value per PEI for each participant.

To investigate if the sensory threshold differed among the six PEIs of the control group, the Kruskal-Wallis test was applied, followed by Dunn's Multiple Comparison Test to compare the six PEIs with each other—alfa = 0.05.

#### Control vs. Patients

The Mann-Whitney test was applied to compare PEIs between control participants and BPI patients (control × injured; control × uninjured upper limbs). For each patient, threshold values for all PEIs of the uninjured limb were normalized to the median values of the control group and these normalized data were used to calculate Spearman correlation coefficients employing the patients' age and the time interval between the injury and the assessment (T2 from Table 1).

To investigate the effect of the side of the injury, patients were separated into two groups comprising BPI in the dominant right (*n* = 7) or non-dominant left limb (*n* = 8). BPI03 and BPI04 were excluded from the sample because they were ambidextrous and left handed, respectively (Table 1). The Mann-Whitney test was performed to compare the threshold values of the uninjured side of both patient groups (dominant and non-dominant injury) with those of the control group. In addition, a K-means clustering algorithm was applied to the BPI group to separate patients into two groups as a function of their sensory impairment (k = 2). Thus, based on their PEI threshold values on the injured side, patients were grouped into more or less severely affected. Sensory thresholds of the uninjured side of both patient groups were then compared with the control group using the Mann-Whitney test. This analysis aimed to investigate if different levels of sensory deficits in the injured side would result in different patterns of sensory changes in the uninjured side.

## Results

### Control Values for Absolute Touch Threshold—“Typical” Index

**Figure 2:**
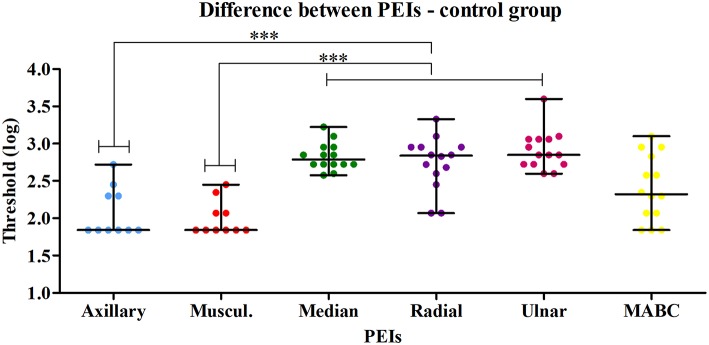
Comparison between the six PEIs of the control group revealed a significant difference (Kruskal-Wallis test—*p* < 0.0001). Dunn's Multiple Comparison Test (alfa = 0.05) applied to compare all pairs of PEIs revealed that the most proximal PEIs (axillary and musculocutaneous) had lower thresholds than the distal PEIs (median, radial and ulnar). ^***^ *p* < 0.05 at the Dunn's Multiple Comparison Test.

A typical index per PEI was calculated for control subjects. The comparison among the six PEI threshold values in the control group revealed a significant difference (*p* < 0.0001). Dunn's Multiple Comparison Test (alfa = 0.05) revealed that the most proximal PEIs had lower threshold values than the most distal ones (Figure 2). Median (25th and 75th percentiles) PEI threshold values are presented in Table 2.

**Table 2:** Median (Q1–Q3) threshold values for the control group and the injured and uninjured upper limbs of the BPI patients.

**PEIs**	**Control (*n* = 14)**	**Injured (*n* = 17)**	**Uninjured (*n* = 17)**
Axillary	1.84 (1.84–2.30)	6.02 (4.84–6.20)^***^	1.84 (1.84–2.30)
Musculoc.	1.84 (1.84–2.07)	6.20 (2.45–6.20)^***^	1.84 (1.84–1.84)
Median	2.79 (2.72–2.95)	4.65 (3.69–6.20)^***^	3.06 (2.95–3.60)^*^
Radial	2.84 (2.56–2.95)	4.65 (3.42–6.20)^***^	3.06 (2.85–3.69)^*^
Ulnar	2.85 (2.72–3.06)	4.11 (3.33–6.20)^**^	3.60 (2.85–3.69)^*^
MABC	2.32 (2.01–2.86)	4.11 (2.83–6.20) ^**^	2.85 (2.45–3.60)^*^

* *p ≤ 0.05*,

** *p ≤ 0.001*,

*** *p ≤ 0.0001 compared to the control group (n = 14)*.

### Control vs. Injured Side

**Figure 3:**
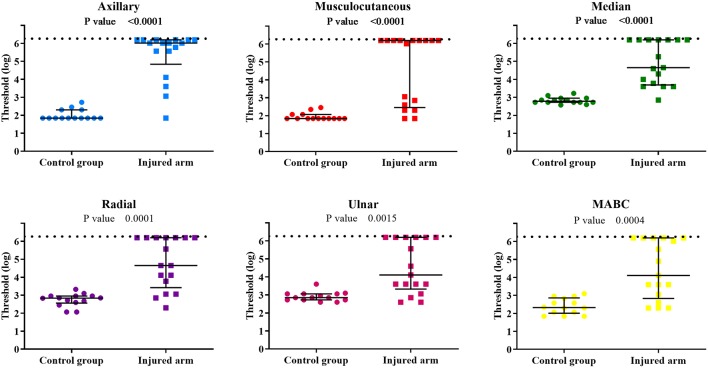
Individual sensory threshold values in the 6 PEIs of the control group compared to the BPI patients' injured upper limb. Lines represent the median values of each group with the interquartile ranges. *P*-values of the statistical difference between groups (control × injured) are presented in each graph. The broken line represents the top limit of the set of monofilaments used (6.20). ● = control (*n* = 14) and ■ = BPI patients (*n* = 17).

The comparison between the control group and the BPI patients' injured side showed a clear difference for all evaluated PEIs (Figure 3). It confirms the important sensory deficit in the latter group. Touch thresholds on the injured side were highly variable among patients, ranging from 1.84 to 6.20 (Table 2). For several patients, even the thickest monofilament was not detectable in some PEIs.

### Control vs. Uninjured Side

**Figure 4:**
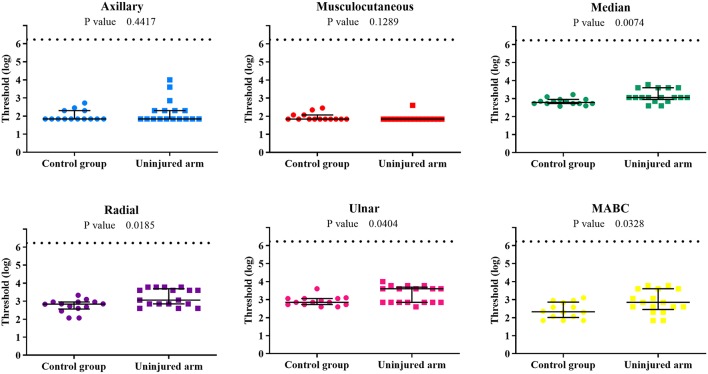
Individual sensory threshold values of the 6 PEIs of the control group compared to the BPI patients' uninjured upper limb. Lines represent the median values and the interquartile ranges of each group. *P*-values of the difference between groups (control × uninjured) are presented in each graph. The broken line represents the top limit of the set of monofilaments (6.20). ● = control (*n* = 14) and ■ = BPI patients (*n* = 17).

The comparison between the control group and the patients' uninjured side is shown in Figure 4. Statistical analysis showed differences for the median, radial, ulnar, and MABC PEIs, indicating that the uninjured side is also affected by the BPI. Moreover, sensory threshold values from the patients uninjured limb were globally considerably more variable than those of the control group, with several patient values lying above the control group range (Table 2).

Spearman correlation coefficients analysis showed no significant correlation between the normalized values of each uninjured PEI and patient age or for the time interval between the injury and the assessment (*p* > 0.1).

### Side of Injury and Sensory Deficits in the Uninjured Side

After excluding the left handed and the ambidextrous patients we found that patients with BPI in the dominant right limb presented higher threshold values for the uninjured side compared to the control group at the median (*p* = 0.0456), radial (*p* = 0.0096) and MABC (*p* = 0.0078) PEIs (axillary: *p* = 0.0732; musculocutaneous: *p* = 0.6531; ulnar: *p* = 0.0628). Conversely, patients with BPI in the non-dominant left limb showed no PEI threshold difference from those of the control group (axillary: *p* = 0.3762; musculocutaneous: *p* = 0.1157; median: *p* = 0.0568; radial: *p* = 0.2722; ulnar: *p* = 0.4035; MABC: *p* = 0.6539). Table 3 presents median threshold values of both groups. With respect to the ambidextrous and the left handed patients we cannot take any conclusion regarding the small sample size.

**Table 3:** Median (Q1–Q3) threshold values for the injured and uninjured upper limbs after a BPI in the dominant and non-dominant limb.

	**Dominant (*n* = 7)**		**Non-dominant (*n* = 8)**	
**PEIs**	**Injured**	**Uninjured**	**Injured**	**Uninjured**
Axillary	6.02 (4.11–6.20)^***^	2.30 (1.84–3.60)	6.02 (5.62–6.20)^**^	1.84 (1.84–1.84)
Musculoc.	2.85 (1.84–6.20)^*^	1.84 (1.84–1.84)	6.20 (2.75–6.20)^***^	1.84 (1.84–1.84)
Median	3.78 (3.60–5.26)^**^	3.06 (2.85–3.60)^*^	6.20 (4.61–6.20)^***^	3.06 (2.90–3.06)
Radial	4.65 (2.85–5.57)^*^	3.60 (2.85–3.60)^*^	6.20 (3.863–6.20)^**^	2.95 (2.60–3.73)
Ulnar	3.60 (2.85–4.60)^*^	3.60 (2.85–3.60)^*^	5.88 (3.60–6.20)^**^	2.85 (2.85–3.73)
MABC	3.06 (2.30–4.90)^*^	3.06 (2.85–3.60)	6.11 (3.73–6.20)^***^	2.60 (1.95–2.60)

* *p ≤ 0.05*,

** *p ≤ 0.001*,

*** *p ≤ 0.0001 compared to the control group (n = 14)*.

### Severity of Injury and Sensory Deficits in the Uninjured Side

A K-means clustering algorithm applied to the BPI group (k = 2) resulted in the formation of two groups: one with less severe sensory impairment (*n* = 10: BPI01, BPI02, BPI03, BPI07, BPI08, BPI09, BPI10, BPI11, BPI13, and BPI14) and the other with more severe BPI impairment of sensory function (*n* = 7: BPI04, BPI05, BPI06, BPI12, BPI15, BPI16, and BPI17). For graphs depicting the patients' individual assessment of both limbs, see Supplementary Figures 1, 2.

The comparison between the control group and the uninjured side of the less severely affected patients (*n* = 10) revealed higher thresholds for patients at the median (*p* = 0.0189), radial (*p* = 0.0081), ulnar (*p* = 0.0253), and MABC (*p* = 0.0187) PEIs (axillary: *p* = 0.6482; musculocutaneous: *p* = 0.3864). Similar results were obtained after excluding the ambidextrous patient from the sample, with higher thresholds for the uninjured side in the less severely affected patients (*n* = 9) compared to the control group (axillary: *p* = 0.5261; musculocutaneous: *p* = 0.4581; median: *p* = 0.0277; radial: *p* = 0.0161; ulnar: *p* = 0.0466; and MABC: *p* = 0.0367).

From this sample of less severely affected patients, six have undergone ulnar to musculocutaneous nerve transfer. For these 6 patients the difference between the uninjured side and the control group was found only for the ulnar nerve (*p* = 0.0332) (axillary: *p* = 0.5500; musculocutaneous: *p* = 0.1745; median: *p* = 0.0791; radial: *p* = 0.1582; MABC: *p* = 0.2452).

The comparison between the uninjured side of the more severely affected patients and the control group showed statistical difference only for the median PEI (*p* = 0.0457) (axillary: *p* = 0.3976; musculocutaneous: *p* = 0.3958; radial: *p* = 0.3109; ulnar: *p* = 0.3434; MABC: *p* = 0.3288). This group comprised 6 right-handed and one left-handed patients. When the left handed patient was excluded from this group, no difference between the uninjured side and the control group was observed (axillary: *p* = 0.7989; musculocutaneous: *p* = 0.1745; median: *p* = 0.1033; radial: *p* = 0.3002; ulnar: *p* = 0.6440; MABC: *p* = 0.4543).

Taken together, these results suggest that more severe injuries are associated with minor sensory dysfunction in the uninjured upper limb. In contrast, for the less severe group, the unaffected upper limb presented a higher number of affected PEIs. Table 4 presents median threshold values of both groups.

**Table 4:** Median (Q1–Q3) threshold values for the injured and uninjured upper limbs of the less and more severe BPI.

	**Less severe (*n* = 10)**		**More severe (*n* = 7)**	
**PEIs**	**Injured**	**Uninjured**	**Injured**	**Uninjured**
Axillary	5.67 (3.98–6.02)^***^	1.84 (1.84–2.30)	6.20 (6.02–6.20)^**^	1.84 (1.84–2.85)
Musculoc.	2.72 (2.18–6.06)^*^	1.84 (1.84–1.84)	6.20 (6.20–6.20)^***^	1.84 (1.84–1.84)
Median	3.89 (3.60–4.61)^**^	3.06 (3.01–3.19)^*^	6.20 (6.20–6.20)^**^	3.06 (2.85–3.60)^*^
Radial	3.94 (3.01–4.65)^*^	3.60 (2.85–3.78)^*^	6.20 (6.20–6.20)^**^	2.85 (2.60–3.60)
Ulnar	3.60 (2.79–3.73)	3.60 (2.85–3.64)^*^	6.20 (6.20–6.20)^**^	2.85 (2.85–3.78)
MABC	3.33 (2.30–3.73)^*^	3.06 (2.52–3.64)^*^	6.20 (6.02–6.20)^**^	2.60 (2.30–2.85)

* *p ≤ 0.05*,

** *p ≤ 0.001*,

*** *p ≤ 0.0001 compared to the control group (n = 14)*.

## Discussion

By applying Semmes-Weinstein monofilaments, we were able to characterize, for the first time, superficial sensibility deficits in points of exclusive innervation (PEI) both in the injured and the uninjured upper limbs in BPI patients as compared to an age-paired and sex-matched control group. The comparison between the control group and the BPI patients' injured side showed higher thresholds for all PEIs in the affected limb. Interestingly, the comparison between the sensory thresholds of the control group and those of the BPI patients' uninjured side also revealed higher thresholds for the median, radial, ulnar and MABC PEIs. After splitting the sample of right handed patients with respect to the dominance of the injured limb, we found higher threshold values for the uninjured side in the BPI patients in the right dominant limb compared to the control group at the median, radial and MABC PEIs. On the other hand, in patients with BPI in the left non-dominant arm, this effect was absent. Using the K-means clustering algorithm, the patient sample was then split into two groups based on the threshold values of the injured side. Thresholds of the uninjured side of the resulting two groups of patients were compared to those of the control group. Less severely affected BPI patients (*n* = 10) had higher thresholds for the median, radial, ulnar and MABC PEIs. Conversely, more severe BPI patients showed higher thresholds only for the median PEI. These results suggest that both the laterality and the degree of sensory impairment of the injured arm associate to higher sensory thresholds in the uninjured side. These findings expand the current knowledge on sensory changes after peripheral nerve lesions, providing novel approaches to clinical evaluation and also opening up new possibilities for the study of central reorganization after peripheral injury.

### “Typical” Threshold Values for the Upper Limb

In this study, it was possible to establish the control group's “typical” threshold for each PEI. The lack of difference between the sensory thresholds for the left and right upper limb in the control subjects corroborates previous findings (28). A “typical” threshold value has been previously described in healthy subjects (29). Many studies have employed this threshold value to evaluate all dermatomes of the upper limb (hand/arm/forearm) (11, 12, 14). Nevertheless, we found lower threshold values in proximal PEIs (axillary and musculocutaneous) than in distal PEIs (median, radial and ulnar). One possible explanation for this discrepancy may be related to the hairiness of the evaluated skin. Proximal PEIs are in hairy skin regions while the distal ones are in glabrous skin (30). The hair follicle shaft has collars of mechanoreceptor terminals, including at least three low threshold mechanoreceptor subtypes (31). Therefore, hair deflection might be associated with a lower threshold in the proximal PEIs. In this case, applying the normative threshold value of 2.85, which is higher than the “typical” threshold found for this population, to assess the entire upper limb could induce the detection of false negative sensory deficits.

### Threshold Values in the Injured Upper Limb After a BPI

The assessment of the injured upper limb of the BPI patients at the six selected PEIs agreed with their clinical diagnoses. Indeed, in the majority of our sample BPI had been documented as affecting mainly the superior and middle trunk of the brachial plexus. Accordingly, axillary, musculocutaneous and the median PEIs presented the highest sensory thresholds when compared to the control group. The huge variability amongst threshold values found for most PEIs in the injured limb (Figure 2) can be attributed to the highly variable degree of severity that is commonly seen for BPI (1).

### Unilateral BPI Induces Bilateral Touch Threshold Impairment

BPI led to increased tactile threshold in specific PEIs of the uninjured upper limb. These results are in agreement with a variety of deafferentation models that also found changes in sensory processing in the uninjured limb (18–23, 32). Both in burn patients (22, 23) and lower limb amputees (20), the assessment of cutaneous threshold presented higher values for the uninjured limb as compared to controls.

The sensory impairment in the uninjured limb found in BPI patients suggests that BPI leads to central modifications in the hemisphere contralateral to the uninjured limb, as shown in different models of deafferentation (33–41). As reviewed by Wall et al. (42), chronic nerve injuries (peripheral nerve injury, root injury, spinal injury, and amputation) in humans and primates promote not only cortical but also subcortical changes at all levels of the somatosensory system. In animal models, unilateral injuries also lead to bilateral changes in the somatosensory ascending pathway (43–46).

For contralateral alterations to be reflected in the ipsilateral hemibody there must be an interhemispheric transfer of information at some level in the brain. Sensory input is ordinarily processed in the contralateral primary somatosensory cortex but there is also to some extent an ipsilateral cortical response to peripheral stimulation (47, 48). As shown by Iwamura et al. (47), this ipsilateral response seems to depend on the opposite hemisphere via transcallosal connections between homotopic areas. In addition, as described by Preuss and Goldman-Rakic (49) in primates, the midline thalamic nuclei project bilaterally to the prefrontal cortex, an important path of top-down regulation of attention to a given stimulus (50). Thus, the altered ascending information coming from the injured limb could lead to reduced activity in the intralaminar nuclei and reduce the attentional network associated with the detection of ascending tactile stimuli, both for the injured and the uninjured limb.

### BPIs of the Dominant Right Limb Are Linked to a Large Sensory Impairment in the Uninjured Side

When the injury occurred in the dominant right limb, higher sensory thresholds were observed in the non-dominant left uninjured limb. Thus, higher thresholds were found for the uninjured limb when the limb was the dominant one. With respect to the ambidextrous and the left handed patients we cannot take any conclusion due to the small sample size.

A behavioral study of tactile perception after bilateral hand transplant showed that the somatosensory perception of the right hand was largely reduced when the right side of the face was concurrently stimulated. The same was not true for the left side (51). After a bilateral hand allograft, Vargas et al. (52) showed that the motor cortical reorganization was faster and more extensive for the non-dominant hand than for the dominant hand. One possibility raised by these authors was that the hemisphere contralateral to the dominant hand is more “hardwired” (and thus less plastic) in the context of a bilateral hand allograft. Interestingly, the amputation of the dominant upper limb elicits more errors and slower responses in motor imagery and handedness judgment tasks (53).

Greater cortical functional reorganization is observed in patients with BPI when the dominant limb is affected as compared to the non-dominant limb (54). This suggests that a relatively more extensive adaptive process may occur following an injury to the dominant hand. The long-standing loss and/or disuse of the dominant limb may degrade the sensorimotor efficiency of both the dominant and the non-dominant upper limb. If this is correct, then it is possible that a dominant side BPI has a greater impact over sensorimotor representations, with reduced plasticity in the hemisphere contralateral to the dominant hand.

### Reduced Sensory Impairments in the Injured Side Are Linked to Greater Sensory Impairment in the Uninjured Side

The group of patients with less severe sensory impairment presented a higher number of affected PEIs in their uninjured upper limb, while the more severe lesions were associated with lower sensory dysfunction in the uninjured upper limb. These results suggest that the severity of injury is inversely related to the impaired threshold detection observed in the uninjured upper limb. One possibility is that the less severe injury is associated with a higher sensorimotor disorganization in the hemisphere contralateral to the injured limb. This, in turn, would affect the representations of the uninjured limb to a greater extent. Indeed, Jain et al. (55) showed in owl monkeys that an incomplete unilateral cervical (C3–C4) dorsal column section leads to expanded cortical representations in the contralateral cortical somatosensory area 3b. Interestingly, the remaining parts of the hand in the deafferented cortical areas present larger receptive fields and abnormal response properties compared to complete lesions. It was also shown that the combination of median+radial nerve injury leads to far more silent regions in the primary somatosensory cortex (S1) than that of the median+ulnar lesion (56–58). These results suggest that both the type and extent of peripheral lesion matter when it comes to the cortical reorganization of S1 topographical maps. The manner by which these changes reflect modifications of the representation of the unaffected limb is unknown. The abnormal reorganization taking place in the less severe injuries can thus contribute to higher touch thresholds in the contralateral hemibody due to changes in interhemispheric communication of cortical maps (40, 41).

The group of patients classified as more severely impaired corresponded to those with extended lesions (superior, medium, and inferior trunks, SMI), most of them having undergone an intercostal to musculocutaneous nerve transfer. This surgery has already been proven to produce changes in the biceps cortical representation (59, 60). However, the small sample of patients with this type of surgery recruited in the present study precluded any further analysis on the effect of this nerve transfer upon sensory threshold impairment in the uninjured limb.

The group of less severely affected patients comprised those having undergone ulnar to musculocutaneous nerve transfer. Contrasting with the prevailing effect of higher threshold values found for the uninjured side, for these patients the difference between the uninjured side and the control group was found only for the ulnar nerve. As discussed above, a less severe injury would be associated with a higher sensorimotor disorganization in the hemisphere contralateral to the injured limb. This in turn would lead to a change in the cortical representation of the uninjured limb. In this context, the ulnar to musculocutaneous nerve transfer, already associated to functional improvement of the affected limb (61) and possibly of cortical representation restoration (62), could play a role by reducing the tactile threshold impairment of the uninjured limb through callosal modulatory effects (38).

## Conclusion

This is the first report of superficial sensibility deficit in both injured and the uninjured upper limbs in BPI patients. Furthermore, the higher sensory deficits in the uninjured side were associated to BPI of the dominant limb and the lower severity of sensory deficits in the injured side. These findings corroborate previous results reported after other peripheral injuries such as in amputees and suggest a superordinate model of representational plasticity occurring bilaterally in the brain after unilateral peripheral deafferentation. Indeed, peripheral nerve lesion causes continuous, time-dependent adaptation in the cortical network (63, 64). Among the limitations of the current study we highlight the small sample size as well as its heterogeneity regarding the lesion extent, the occurrence and type of surgical intervention and the time elapsed from lesion. Expanding the knowledge on sensory changes after peripheral nerve lesions might provide novel approaches to understand and treat BPI.

The raw data supporting the conclusions of this manuscript will be made available by the authors, without undue reservation, to any qualified researcher.

## Ethics Statement

Participants were informed about the experimental procedures and provided written informed consent to participate in the study, which was approved by the ethics committee of the Institute of Neurology Deolindo Couto—UFRJ, Brazil (number: 298.925 from June 10th, 2013), and was in accordance with the declaration of Helsinki.

## Author Contributions

BR: conception, data collection, analysis, and interpretation and drafting. MR: data collection, analysis, and interpretation and drafting. AS: conception and data collection. FE and CV: conception, data analysis, and interpretation and revision.

### Conflict of Interest Statement

The authors declare that the research was conducted in the absence of any commercial or financial relationships that could be construed as a potential conflict of interest.

## Supplementary Material

Supplementary Figure 1 Individual sensory threshold values in the 6 PEIs of each BPI patients' injured (dark gray) and uninjured (light gray) upper limbs of the more severe group. The black line represents the median values of the control group (*n* = 14). The top dashed line represents the higher threshold value possible to assess with our filaments set (6.20). ϕ = PEIs in which the subjects were unable to feel even the thickest filament assessed (6.20).

Supplementary Figure 2 Individual sensory threshold values in the 6 PEIs of each BPI patients' injured (dark gray) and uninjured (light gray) upper limbs of the less severe group. The black line represents the median values of the control group (*n* = 14). The top dashed line represents the higher threshold value possible to assess with our filaments set (6.20). ϕ = PEIs in which the subjects were unable to feel even the thickest filament assessed (6.20).

Supplementary Table 1 Calibration measures of the set of 20 SWM used. Forces in grams (g) and the log of the force (log) (10 × force in mg) needed to bend the filament (90° to the skin) in a “C” shape according to the manufacturer (Manufacturer's information) and after filaments calibration (Mean of the Calibrations).

## Abbreviations

BPI: Brachial plexus injury
MABC: medial antebrachial cutaneous nerve
PEI: Point of exclusive innervation
SWM: Semmes-Weinstein monofilaments.
