# Supplementary figures and images for: Unilateral Brachial Plexus Lesion Impairs Bilateral Touch Threshold
Source: Front Neurol. 2019 Aug 13;10:872. doi: 10.3389/fneur.2019.00872 (PMC6700256; doi:10.3389/fneur.2019.00872)

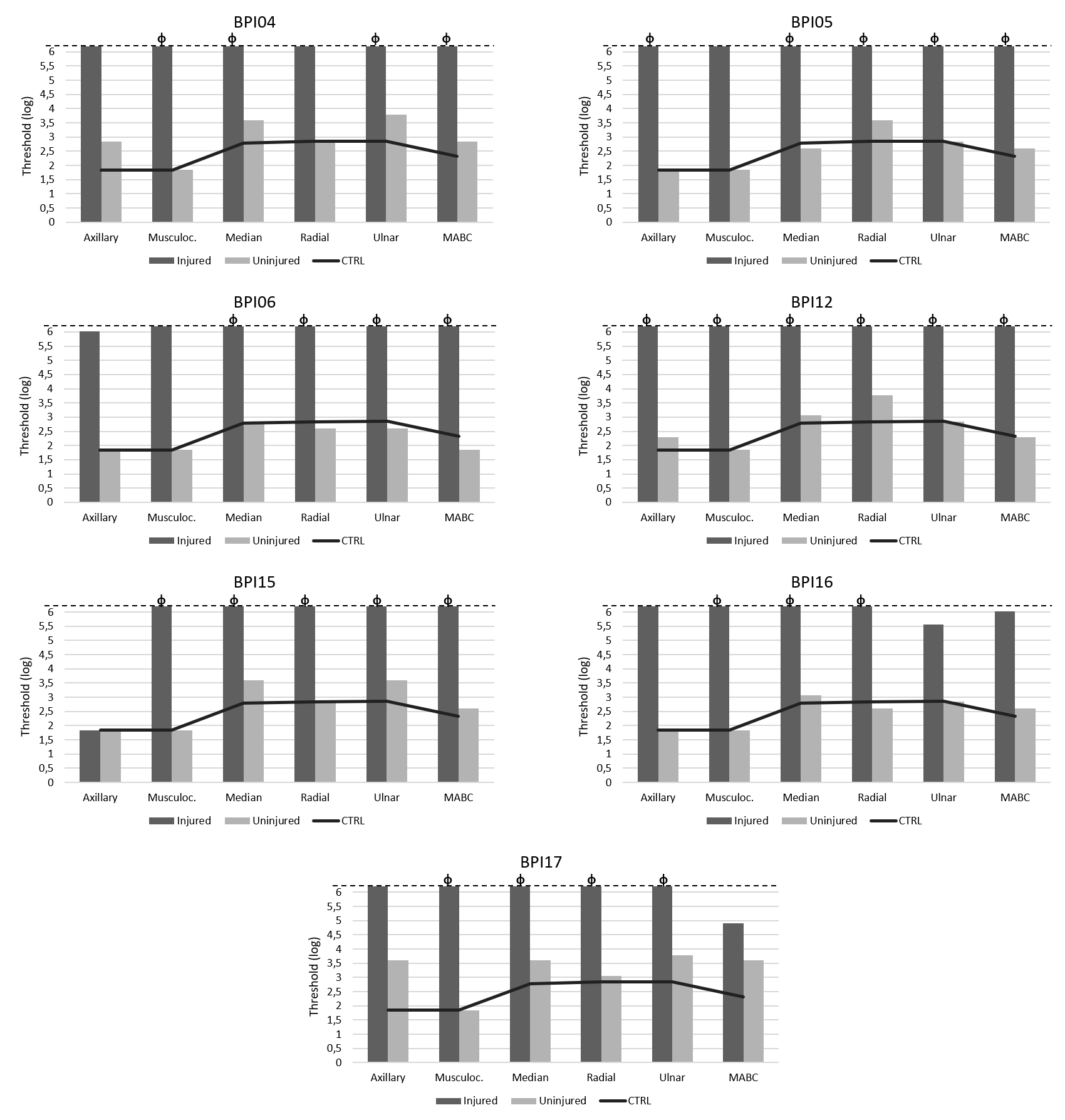

Supplement: Supplementary Figure 1 — Individual sensory threshold values in the 6 PEIs of each BPI patients' injured (dark gray) and uninjured (light gray) upper limbs of the more severe group. The black line represents the median values of the control group (n = 14). The top dashed line represents the higher threshold value possible to assess with our filaments set (6.20). ϕ = PEIs in which the subjects were unable to feel even the thickest filament assessed (6.20). [file Image_1.TIF]

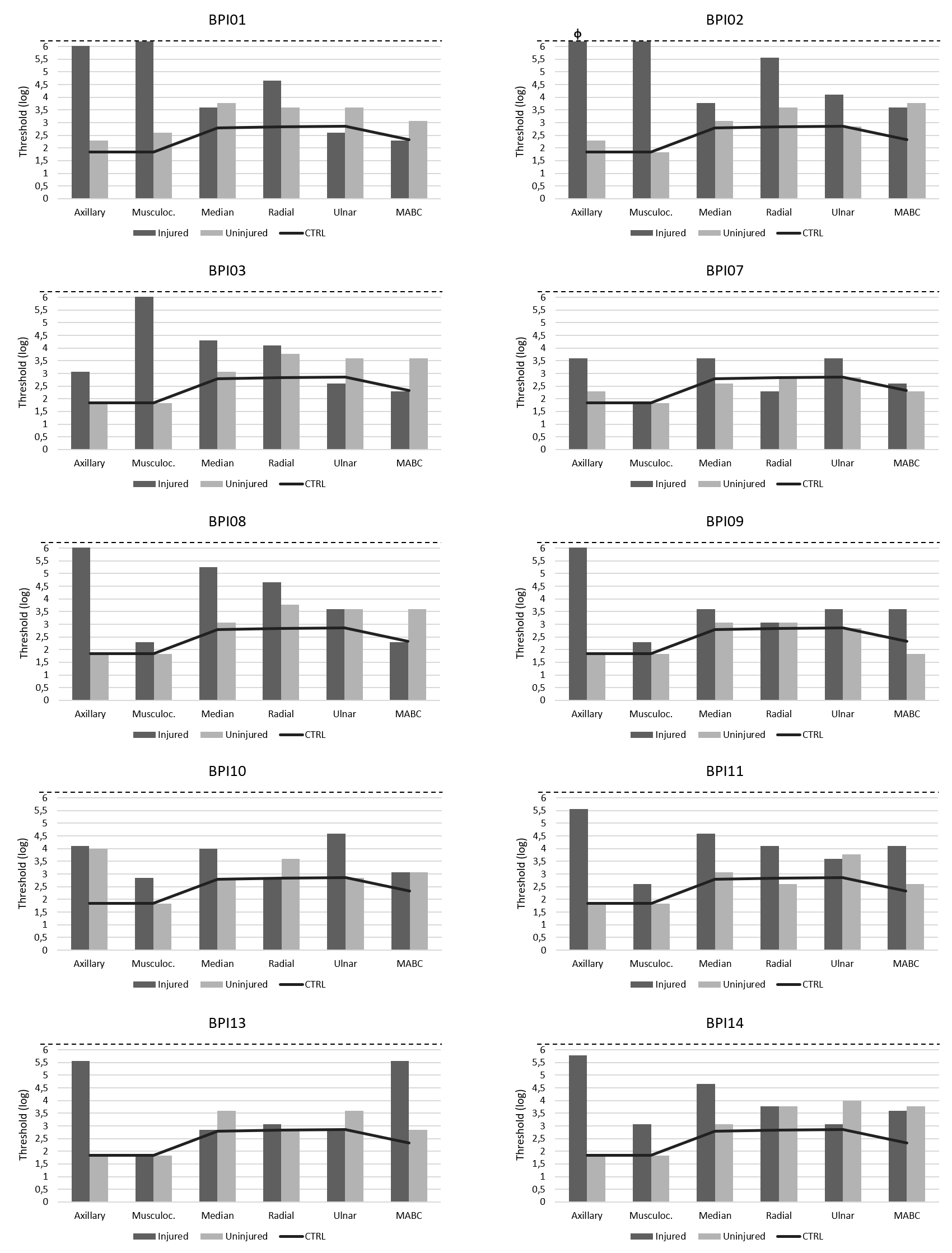

Supplement: Supplementary Figure 2 — Individual sensory threshold values in the 6 PEIs of each BPI patients' injured (dark gray) and uninjured (light gray) upper limbs of the less severe group. The black line represents the median values of the control group (n = 14). The top dashed line represents the higher threshold value possible to assess with our filaments set (6.20). ϕ = PEIs in which the subjects were unable to feel even the thickest filament assessed (6.20). [file Image_2.TIF]
