# Supplementary material for: Unilateral Brachial Plexus Lesion Impairs Bilateral Touch Threshold
Source: Front Neurol. 2019 Aug 13;10:872. doi: 10.3389/fneur.2019.00872 (PMC6700256; doi:10.3389/fneur.2019.00872)
Supplement: Supplementary Table 1 — Calibration measures of the set of 20 SWM used. Forces in grams (g) and the log of the force (log) (10 × force in mg) needed to bend the filament (90° to the skin) in a “C” shape according to the manufacturer (Manufacturer's information) and after filaments calibration (Mean of the Calibrations). [file Table_1.DOCX]

Supplementary Material

**Unilateral brachial plexus lesion impairs bilateral touch threshold**

**Bia Lima Ramalho^*^, Maria Luíza Rangel, Ana Carolina Schmaedeke, Fátima Smith Erthal, Claudia D. Vargas**

*** Correspondence:** Bia Lima Ramalho: ramalhobsl@gmail.com

**1. Supplementary Data**

- 1. **Monofilaments calibration**

The set of 20 SWM (Bioseb, Vitrolles, France), according to the manufacturer’s specifications, comprised monofilaments ranging from 0.008g to 300g or, expressed in log (10 x F; with F= force in milligrams), 1.90 to 6.48 (Manufacturer’s information – Supplementary Table 1). In order to assess whether the actual force needed to bend the filaments was in accordance with that specified by the manufacturer, each monofilament was assessed using an analytical balance (Shimadzu Corp., Kyoto, Japan). Each filament was applied over the balance plate by the same experimenter who recorded the results. The procedure was repeated 3 times for each filament and the average value was used to determine each filament force (g). The calibration procedure was performed twice, before the beginning of sensory threshold data collection (calibration 1) and again after approximately half of the participants had been tested (calibration 2). The values obtained for the two calibrations in different periods were similar, but for some monofilaments our calibrations differed from those provided by the manufacturer (see supplementary Table 1). We decided to use the values found in our calibration. The evaluation was based on the log of each monofilament’s mean force after the 6 calibration trials (3 at calibration 1, and 3 at calibration 2) (Mean of the Calibrations) (Supplementary Table 1).

1. **Supplementary Figures and Table**
   1. **Supplementary Table**

**Supplementary Table S1 –** Calibration measures of the set of 20 SWM used. Forces in grams (g) and the log of the force (log) (10 x force in mg) needed to bend the filament (90° to the skin) in a "C" shape according to the manufacturer (Manufacturer’s information) and after filaments calibration (Mean of the Calibrations).

| Manufactor Informations | | Mean of 2 Calibrations | |
| --- | --- | --- | --- |
| g | log | g | log |
| 0.008 | 1.90 | 0.007 | 1.84 |
| 0.02 | 2.30 | 0.02 | 2.30 |
| 0.04 | 2.60 | 0.04 | 2.60 |
| 0.07 | 2.85 | 0.07 | 2.85 |
| 0.16 | 3.20 | 0.12 | 3.06 |
| 0.4 | 3.60 | 0.4 | 3.60 |
| 0.6 | 3.78 | 0.6 | 3.78 |
| 1 | 4.00 | 1 | 4.00 |
| 1.4 | 4.15 | 1.3 | 4.11 |
| 2 | 4.30 | 2 | 4.30 |
| 4 | 4.60 | 4 | 4.60 |
| 6 | 4.78 | 4.5 | 4.65 |
| 8 | 4.9 | 7 | 4.85 |
| 10 | 5.00 | 8 | 4.90 |
| 15 | 5.18 | 12 | 5.08 |
| 26 | 5.41 | 18 | 5.26 |
| 60 | 5.78 | 37.5 | 5.57 |
| 100 | 6.0 | 60.5 | 5.78 |
| 180 | 6.26 | 104 | 6.02 |
| 300 | 6.48 | 160 | 6.20 |
